# Research Progress of Artificial Intelligence Image Analysis in Systemic Disease-Related Ophthalmopathy

**DOI:** 10.1155/2022/3406890

**Published:** 2022-06-24

**Authors:** Yuke Ji, Nan Chen, Sha Liu, Zhipeng Yan, Hui Qian, Shaojun Zhu, Jie Zhang, Minli Wang, Qin Jiang, Weihua Yang

**Affiliations:** ^1^The Laboratory of Artificial Intelligence and Bigdata in Ophthalmology, Affiliated Eye Hospital, Nanjing Medical University, Nanjing, China; ^2^School of Information Engineering, Huzhou University, Huzhou, China; ^3^Advanced Ophthalmology Laboratory (AOL), Robotrak Technologies, Nanjing, China; ^4^First Affiliated Hospital of Huzhou University, Huzhou, China

## Abstract

The eye is one of the most important organs of the human body. Eye diseases are closely related to other systemic diseases, both of which influence each other. Numerous systemic diseases lead to special clinical manifestations and complications in the eyes. Typical diseases include diabetic retinopathy, hypertensive retinopathy, thyroid associated ophthalmopathy, optic neuromyelitis, and Behcet's disease. Systemic disease-related ophthalmopathy is usually a chronic disease, and the analysis of imaging markers is helpful for a comprehensive diagnosis of these diseases. Recently, artificial intelligence (AI) technology based on deep learning has rapidly developed, leading to numerous achievements and arousing widespread concern. Presently, AI technology has made significant progress in research on imaging markers of systemic disease-related ophthalmopathy; however, there are also many limitations and challenges. This article reviews the research achievements, limitations, and future prospects of AI image analysis technology in systemic disease-related ophthalmopathy.

## 1. Introduction

The term artificial intelligence (AI) was proposed by John McCarthy in 1956. It is a new technical science that studies and develops theories, methods, technologies, and application systems used to simulate, extend, and expand human intelligence. Since it was proposed in 1956, AI has played a significant role in image recognition, data mining, and language processing, showing infinite potential. In recent years, AI technology has developed rapidly and has been widely employed in numerous fields. Among them, AI image analysis technology has made great progress and has played a significant role in research and application in the medical field, particularly in ophthalmology [1]. Because the development and anatomy of the eye are closely related to the whole body, systemic diseases such as diabetes, hypertension, hyperthyroidism, and autoimmune diseases can cause eye diseases. Some eye manifestations are characteristic. By examining the ocular structures such as the fundus and orbit, not only can various ophthalmic diseases be diagnosed but also the condition of systemic diseases involving the eyes can be judged. Recently, the main research and application direction of AI technology in ophthalmology is the use of deep learning to study and analyze eye images to assist in the diagnosis of ophthalmopathy. Applying AI image analysis based on deep learning to the automatic analysis of eye images is crucial to diagnose the corresponding ophthalmopathy and judge the condition of related systemic diseases (such as diabetes, hypertension, and hyperthyroidism). Presently, AI image analysis technology has led to significant achievements in research on systemic disease-related ophthalmopathy. This paper mainly reviews the research progress of AI image analysis technology in systemic disease-related ophthalmopathy in recent years.

**Figure 1:**
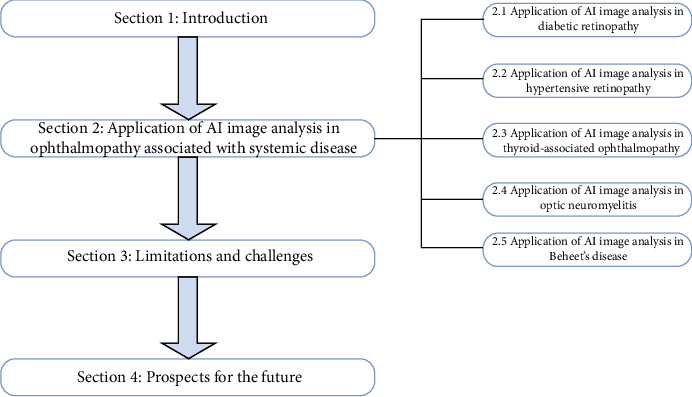
The basic framework of this review paper.

This review paper is divided into four parts. Firstly, the first part introduces the basic research status of AI technology in systemic disease-related ophthalmopathy. Secondly, the second part introduces the research progress of AI technology in systemic disease-related ophthalmopathy, such as diabetic retinopathy, hypertensive retinopathy, thyroid-associated ophthalmopathy, neuromyelitis optica, and Behcet's disease. Thirdly, the third section discusses the limitations and challenges of medical application of AI technology. Finally, the fourth section discusses the prospect of medical application of AI technology, as shown in Figure 1.

## 2. Application of AI Image Analysis in Ophthalmopathy Associated with Systemic Disease

### 2.1. Application of AI Image Analysis in Diabetic Retinopathy

Diabetic retinopathy (DR) is one of the main complications of diabetic microangiopathy. It is a chronic, progressive, and potentially harmful retinal microvascular disease that is associated with persistent hyperglycemia. DR is the most common retinal vascular disease [2] and is also the retinal vascular disease with the highest blindness rate [3]. In people with diabetes, the main factors affecting the occurrence and development of DR are the course of the disease and the level of blood glucose control [4]. Patients with DR may not have any symptoms in the early stages of the disease and may experience varying degrees of vision loss when the lesions involve the macula. According to disease severity, DR is divided into nonproliferative diabetic retinopathy (NPDR) and proliferative diabetic retinopathy (proliferative diabetic retinopathy, PDR) [5]. The main fundus lesions in patients with DR include retinal microhemangioma, retinal hemorrhage, rigid exudation, cotton velvet spot, retinal edema, retinal neovascularization, and serious complications such as vitreous hemorrhage, traction retinal detachment, neovascular glaucoma, and macular edema. These complications seriously threaten the vision of patients and even lead to blindness. PDR and macular edema are the main causes of severe visual loss in patients with DR [6]. Presently, the main treatment modalities for patients with DR include drug therapy, anti-VEGF drug therapy, laser photocoagulation, and vitrectomy. For DR patients with macular edema, vitreous injection of anti-VEGF drugs can significantly improve visual acuity [7]; for patients with PDR, panretinal laser photocoagulation can effectively alleviate the decline in visual acuity [8]. At present, DR has become one of the main causes of blindness in the world [9], seriously affecting the quality of life of patients. Studies have shown that approximately 90.0% patients with DR can avoid severe vision loss through early diagnosis of DR and timely and effective treatment [10]. Therefore, early detection and treatment of DR can control the condition in most patients and reduce and avoid the occurrence of blindness, which is of great significance.

Gulshan et al. [11] developed a deep convolution neural network based on deep learning to assist in the clinical diagnosis and grading of diabetic retinopathy and diabetic macular edema. In this study, the neural network was trained using a dataset containing 128,175 retinal images, and the neural network was tested and verified using the EyePACS-1 dataset (including 9963 retinal images) and Messidor-2 dataset (including 1748 retinal images). The results indicated that on the EyePACS-1 dataset, the area under the curve (AUC) value was 0.991, the sensitivity was 90.3%, and the specificity was 98.1%; on the Messidor-2 dataset, the corresponding values were 0.990, 87.0%, and 98.5%. Ai et al. [12] constructed an algorithm based on the deep ensemble learning and attention mechanism that could detect HR. Simultaneously, to improve the accuracy of the algorithm, the author created a complete detection model DR-IIXRN (composed of Inception V3, Xception, ResNeXt101, and NASNetLarge). In this study, a total of 945 fundus images were collected, of which 133 were randomly selected for testing, and the remaining were used for training. The experimental results show that, compared with the traditional detection algorithm, the AUC value and accuracy of this algorithm were as high as 95% and 92%, respectively. Bhardwaj et al. [13] constructed a quadrant ensemble-automated DR grading model based on the InceptionResnet-V2 depth neural network. The accuracy of the model was improved through methods such as histogram equalization, optic disk location, and quadrant segmentation. They used the open fundus datasets DRIVE, STARE, and DIARETDB1 to train and test the model. The final results show that the accuracy of the model is as high as 93.33%, which is 13.58% higher than that of the classical Inception-V3 CNN model. Based on Inception-V3, Li et al. [14] proposed a deep learning algorithm based on AI, which was used to detect DR. In this study, 71,043 fundus image photos were used for training and testing of the algorithm, and 35,201 fundus image photos were used for external verification. The results showed that the AUC value, sensitivity, and specificity of the algorithm were 0.989, 97.0%, and 91.4%, respectively.  Table 1 summarizes the DR diagnosis model based on deep learning method in the above research.

Based on the above research results, AI image analysis technology shows great potential in clinical detection, auxiliary diagnosis, staging, and other aspects of DR. At present, for diabetic patients, the timeliness of DR screening, the quality of screening, and medical affordability are important factors to prevent blindness. Therefore, if AI technology can be fully applied to clinical work, it will greatly alleviate the work pressure of doctors, improve work efficiency, and reduce the burden of medical treatment of DR patients; at the same time, it is of great significance for early screening and diagnosis of DR in patients with diabetes, which can greatly reduce the blindness rate of patients with DR.

### 2.2. Application of AI Image Analysis in Hypertensive Retinopathy

According to the international standard for hypertension, hypertension is diagnosed when the systolic blood pressure is greater than or equal to 140 mmHg and diastolic blood pressure is greater than or equal to 90 mmHg [16]. At present, hypertension is a serious epidemiological problem, and approximately 10 million people die from hypertension every year [17]. Hypertensive retinopathy (HR) is a type of retinal angiopathy caused by hypertension. Hypertensive patients experience continuous contraction and tension of systemic arterioles. Long-term hypertension can cause arterial lumen stenosis, subsequently leading to hypertensive arteriosclerosis, resulting in retinal vascular stenosis thinning [18, 19]. In the early stages, the fundus of patients with hypertension may not show any pathological changes. When the systemic arterial pressure increases, the fundus of patients with HR may show pathological changes such as decreased retinal arteriovenous diameter ratio, hypertensive retinal arteriosclerosis, retinal edema and hemorrhage, cotton velvet spot, and optic disk edema [20]. At present, the main treatment modality of HR patients is to control systemic blood pressure within the normal range by oral antihypertensive drugs [21]. If HR patients are complicated with macular edema, vitreous injection of anti-VEGF drugs can significantly improve the visual acuity of patients [22]. The study found that, compared with the more serious fundus lesions in patients with HR, if the early fundus changes can be diagnosed and treated in time, then the fundus changes may be reversible. Because HR is a process of gradual change caused by hypertension, early screening and diagnosis of HR can not only effectively prevent its further development but also lead to the best treatment to obtain the best treatment effect.

Based on the framework of DenseNet, Abbas et al. [23] developed a HYPER-RETINO system by segmenting HR-related lesions to assist in the clinical staging diagnosis of HR. After ten times cross-verification, the sensitivity of the system was 90.5%, the specificity was 91.5%, the accuracy was 92.6%, the **F**1 score was 92%, and the AUC was 0.915. The experimental results show that the system has good applicability for the clinical staging diagnosis of HR. Akbar et al. [24] constructed an intelligent system that can realize automatic detection and classification of HR based on support vector machine and a radial basis function. The system comprises two parts: the first part is used to calculate and analyze the arteriovenous ratio, while the second part is used to analyze the optic disk region. The data verification results showed that the average accuracies of the first part on the INSPIRE-AVR and VICAVR datasets and a local dataset were 95.10%, 95.64%, and 98.09%, respectively, and the average accuracies of the second part on the START and local datasets were 95.93% and 97.50%, respectively. The system can be used as a novel method for automatic detection and grading of HR, which is helpful for clinical diagnosis. Arsalan et al. [25] proposed a dual-residual-stream-based vessel segmentation network (Vess-Net) to segment retinal vessels intelligently to assist in the diagnosis of HR. They used three open vascular segmentation datasets (DRIVE, CHASE-DB1, and STARE) to validate and evaluate the Vess-Net method. The results showed that the sensitivity, specificity, AUC value, and accuracy of Vess-Net on the three datasets were as follows: DRIVE: 80.22%, 98.1%, 98.2%, and 96.55%, respectively; CHASE-DB1: 82.06%, 98.41%, 98.0%, and 97.26%, respectively; and STARE: 85.26%, 97.91%, 98.83%, and 96.97%, respectively. This method can be used to assist in the clinical diagnosis of HR.  Table 2 summarizes the HR diagnosis model based on deep learning method in the above research.

Thus, the above AI image analysis model exhibited good performance for clinical diagnosis and grading of HR and can be used in clinical practice in the future to achieve early detection, diagnosis, and treatment of HR patients.

### 2.3. Application of AI Image Analysis in Thyroid-Associated Ophthalmopathy

Thyroid-associated ophthalmopathy (TAO) is a chronic, multisystem damage disease caused by autoimmune response, which is closely related to thyroid disease. Among adult orbital diseases, the incidence of TAO is the highest [26]. At present, the etiology of the disease is not completely clear, but it has been recognized as an autoimmune or organ immune disease and is closely related to the functional state of the systemic endocrine system. The main clinical manifestations of TAO patients are eyelid sign, exophthalmos, diplopia, eye movement disorder, conjunctival and keratopathy, and optic neuropathy. In severe cases, it can affect the patient's appearance and vision and even lead to blindness [27]. The histopathological changes of TAO are early inflammatory cell infiltration, edema leading to obvious inflammatory reaction, tissue degeneration, and dysfunction caused by fibrosis in the later stages. Early anti-inflammatory therapy using glucocorticoids and radiotherapy can help achieve a good therapeutic effect. After the occurrence of extraocular muscle fibrosis in the later stage, the effect of anti-inflammatory treatment becomes poor, and exophthalmos and strabismus can only be improved by surgical treatment [28].

Lin et al. [29] constructed a deep learning system based on a deep convolution neural network (composed of a convolution layer, pooled layer, and fully connected layer) to distinguish between inactive and active patients of TAO. They also added a nonlinear activation function to optimize the learning system. They collected MRI images of the eyes of 160 patients with TAO. Eighty percent of the images were randomly selected for deep learning system training and verification, and 20% of the images were used for the final test. The experimental results showed that the accuracy, sensitivity, specificity and **F**1 scores of network A were 0.863 ± 0.055, 0.750 ± 0.136, 0.896 ± 0.042, and 0.712 ± 0.121, respectively; the accuracy, sensitivity, specificity, and **F**1 scores of network B were 0.855 ± 0.018, 0.821 ± 0.071, 0.865 ± 0.021, and 0.719 ± 0.040, and the AUC values of the two networks were 0.922. This system can be used to assist in the clinical diagnosis of TAO in the future. Song et al. [30] constructed an AI image analysis model for screening and testing TAO patients based on a ResNet-18 derived network (using 3D-ResNet instead of 2D-ResNet). The orbital CT images of 193 patients with TAO and 715 healthy subjects were used for model training and testing, and the orbital CT images of 49 patients with TAO and 178 healthy persons were used for external verification. The results indicated that in the external verification of the AI image analysis model, the accuracy, sensitivity, and specificity were 0.87, 0.88, and 0.85, respectively, and the AUC value was 0.919. The AI image analysis model may become a new TAO screening tool. Salvi et al. [31] constructed a back propagation neural network model with 17 input parameters for clinical classification and progression prediction of TAO. In this study, the ophthalmic examination results of 242 patients were collected, of which 87 patients were selected to test the clinical classification ability of the model for TAO, and 28 patients were selected to test the ability of the model to predict the progression of TAO. The results indicated that the classification accuracy of the model was 78.3%, and the accuracy of predicting progress was 69.2%. In the authors' view, this model can be used to assist in the clinical classification of TAO and to predict disease progression beginning from the first clinical examination.  Table 3 summarizes the TAO diagnosis model based on deep learning method in the above research.

The above studies confirm that AI technology has achieved some research results in assisting in the clinical diagnosis of TAO. The clinical application of AI image analysis technology will help improve the accuracy of clinical diagnosis of TAO, thereby helping doctors to provide accurate treatment methods for patients.

### 2.4. Application of AI Image Analysis in Optic Neuromyelitis

Optic neuritis (ON) generally refers to various inflammatory lesions involving the optic nerve. Neuromyelitis optica (NMO) and multiple sclerosis (MS) are demyelinating diseases of the central nervous system and are the most common causes of neurological dysfunction in young and middle-aged people [32]. The pathogenesis of MS and NMO is mainly related to various immune-related molecules and immune pathways [33]. NMO mainly selectively involves the optic nerve and spinal cord, and the main clinical manifestations are rapid and severe visual loss in both eyes at the same time or in succession, such as optic disk edema, tortuous dilatation of retinal veins, perioptic exudation, and other fundus changes; recovery of visual function is poor, and most patients suffer from serious visual impairment in both eyes or at least one eye. Treatment is mainly to prevent further disease development; it is particularly important to note that visual dysfunction may only be one of the symptoms of potential systemic diseases, so it is necessary to be referred to the relevant specialties for systemic treatment in time. The clinical manifestations and imaging features of MS and NMO have many similarities. In terms of clinical diagnosis, differentiating between the two diseases is challenging [34, 35]. Approximately 30% of patients with MS are misdiagnosed as having NMO, thus delaying the treatment of the disease and leading to aggravation of the disease [36]. Therefore, it is particularly important to improve the diagnostic accuracy for the two diseases.

Huang et al. [37] collected magnetic resonance images of 116 patients with demyelinating diseases of the central nervous system (including 38 cases of NMO and 78 cases of MS). Based on radiological and clinical features, a multiparameter multivariate random forest (MM-RF) model was developed to assist in the differential diagnosis of NMO and MS. The magnetic resonance images of 86 patients were randomly selected for training the model, and the remaining were used for independent testing of the model. After 10 times cross-validation and independent testing, the results showed that in training, the accuracy of the MM-RF model was 0.849, and the AUC value was 0.826; for testing, the accuracy of the MM-RF model was 0.871, and the AUC value was 0.902. Hagiwara et al. [38] constructed a convolution neural network model based on SqueezeNet to distinguish between NMO and MS. In this study, 35 patients with MS and 18 patients with NMO were enrolled, and left-over cross-validation (leave-one-out cross validation) was used to evaluate the performance of the model. The results showed that the AUC value of the model is 0.859, and the diagnostic accuracies of NMO and MS are 81.1% and 83.3%, respectively. Kim et al. [39] created a 3D convolution neural network model based on ResNeXt to assist in the diagnosis of NMO and MS. In this study, magnetic resonance images of 338 patients (including 213,125 patients with MS and 125 patients with NMO) were collected, and images of 152, 51, and 135 patients were randomly selected for training, verification, and testing of the model, respectively. The final experimental results are as follows: the AUC value of the model was 0.82, and the diagnostic accuracy of MS and NMO was 71.1%.  Table 4 summarizes the NMO diagnosis model based on deep learning method in the above research.

In summary, AI image analysis technology shows satisfactory accuracy in clinical differential diagnosis of NMO and MS. If AI image analysis technology can be fully applied to the process of clinical diagnosis, it will greatly improve the accuracy of differential diagnosis of NMO and MS.

### 2.5. Application of AI Image Analysis in Behcet's Disease

Behcet's disease, first reported by Hulusi Behcet in 1937, is a chronic, persistent, multisystem disease that affects numerous organs [41]. The main pathological change of Behcet's disease is occlusive vasculitis. Simultaneously, the disease is characterized by uveitis, oral ulcer, skin lesions, and pudendal ulcer, among which oral ulcers are the most common [42, 43]. At present, the cause of the disease is not clear because patients with the disease often possess a variety of autoantibodies; therefore, it may be an autoimmune disease. Simultaneously, the disease has an obvious genetic background and is significantly related to HLA-B5 [44]. Studies have found that untreated uveitis in Behcet's disease has a poor prognosis, resulting in a high rate of blindness in patients [45]. At present, the treatment of Behcet's disease complicated with uveitis follows the general principles of uveitis: dilating pupils, antagonizing inflammation, and eliminating etiology.

The Standardization of Uveitis Nomenclature (SUN) Working Group [46] collected the medical records of 248 patients with Behcet's disease complicated with uveitis and 718 patients with other types of uveitis and randomly divided them into training and test sets. Using the machine learning method of multinomial logistic regression for the training set, a standard of uveitis in Behcet's disease was developed to distinguish uveitis from other types of uveitis in Behcet's disease. The standard they developed was verified on the test set, and the results showed that the accuracy was 96.3% on the test set and 94.0% on the training set. The error rate of the differentiation criteria of Behcet's disease complicated with uveitis established in this study was very low, and the standard can be used to assist in clinical diagnosis in the future. Based on the delta-bar-delta training algorithm, Guler and Ubeyli [47] constructed a multilayer perceptron neural network (MLPNN) model for the detection of ocular Behcet's disease. In this study, the Doppler signals of ophthalmic artery were collected from 106 subjects, including 54 patients with ocular Behcet's disease, and the remaining were normal. Fifty-four cases were randomly divided into the training set, the remaining were used as the test set, and 10 cases were randomly selected from the training set as the cross-validation set. The MLPNN model was evaluated by performance indicators and statistical methods, and its accuracy was 96.43% and 93.75%, respectively. Based on the above results, the model is feasible for the detection of ocular Behcet's disease.  Table 5 summarizes the Behcet's disease diagnosis model based on deep learning method in the above research.

To sum up, the AI image analysis model based on the deep learning algorithm showed good performance and practicability for the auxiliary diagnosis of Behcet's disease complicated with uveitis and can be used in clinical diagnosis and treatment in the future.

**Figure 2:**
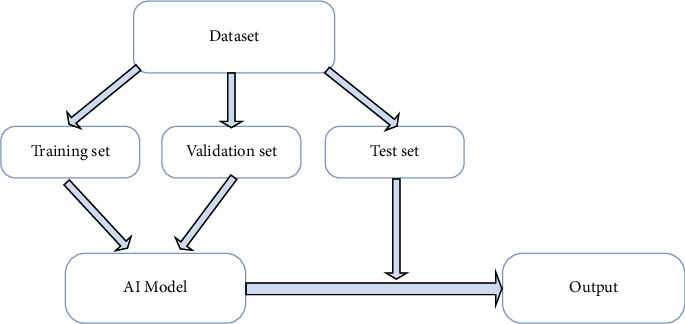
AI model research flow chart.

The flow chart of all the above AI model studies is as follows: firstly, the dataset used for research is randomly divided into training set, validation set, and testing set; then, the training set and validation set are used to train and verify the AI model, and the corresponding improvements and optimizations are made according to the results; finally, the optimized AI model is externally verified with the testing set, and the results are compared with the expert results, so as to obtain the diagnostic performance of the AI model, as shown in Figure 2.

## 3. Limitations and Challenges

At present, in many published studies on AI-aided diagnosis of systemic disease-related ophthalmopathy, although the accuracy and specificity of the AI image analysis model based on deep learning have achieved satisfactory results, there are still some limitations and challenges. The following are the main results: (1) there is a deviation in the datasets of systemic disease-related ophthalmopathy. The training and test datasets used by many AI image analysis models are public datasets, and there are some deviations in these datasets, which lead to a decline in the external applicability of the AI image analysis model and may even magnify these deviations [48]. (2) Deep learning algorithms lack interpretability in clinical applications. Many clinicians do not fully learn and understand the deep learning algorithm, resulting in the “black box phenomenon.” This leads to reduced acceptance of the AI image analysis model, and clinicians may even resist the model [49]. (3) Some patients have prejudice to AI model diagnosis and treatment. Research shows that numerous patients are unwilling or do not accept computer-aided diagnosis but prefer to see a doctor face-to-face, which hinders the clinical application of AI intelligent models because of the lack of trust of patients [50]. After all, medicine is a life science, and it does not rule out that some patients do not trust and do not accept the diagnosis and treatment of artificial intelligence from the very beginning, which is a major obstacle to the development of artificial intelligence in the medical field. (4) Errors in AI models can lead to many medical legal problems. False-positive results may occur in the clinical application of AI image analysis models. If the mistakes of AI image analysis models lead to medical malpractice, then it is unclear who would bear the responsibility and legal consequences of these medical accidents [51]. No one is perfect, and the AI model cannot be perfect, can go wrong, or even cause bigger problems. With the continuous progress of artificial intelligence technology, the AI model may produce results that exceed the designer's design intention. How to determine its behavior? How to strengthen legal monitoring and establish clear ethical norms and legal and regulatory requirements. These are some complex and difficult medical legal problems. (5) Privacy and security of patients: when using patients' clinical data for experiments, clinical data may affect patients' privacy or even cause new social problems, which is a challenge in terms of applying AI image analysis technology [52]. Medical data focus on patients' health, disease status, biological genes, and other information; once leaked, the consequences are unimaginable. Therefore, the health information and privacy protection of patients are also major challenges for artificial intelligence. (6) Legal guarantee of medical application of artificial intelligence technology. In recent years, the rapid development of artificial intelligence technology has brought convenience to all aspects of our life. However, the legislative process of artificial intelligence is relatively slow. The current law does not provide for the rights and obligations of artificial intelligence and the treatment of social hazards, nor does it stipulate the legal provisions, responsibility attribution, and development direction of all aspects of artificial intelligence research and development and development and production process. All these will affect the development and application of artificial intelligence. (7) The social supervision and management system of artificial intelligence technology is not perfect. Artificial intelligence technology involves surpassing human potential, and its development needs to be strictly managed and monitored. At present, the necessary supervision direction and supervision and management system cannot keep up with the development of artificial intelligence technology, which may lead to a large number of ethical and social problems, thus affecting the application of artificial intelligence technology in the medical field. The social supervision and management system of artificial intelligence technology is not perfect. Artificial intelligence technology involves surpassing human potential, and its development needs to be strictly managed and monitored. At present, the necessary supervision direction and supervision and management system cannot keep up with the development of artificial intelligence technology, which may lead to a large number of ethical and social problems, thus affecting the application of artificial intelligence technology in the medical field.

## 4. Prospects for the Future

Although AI technology based on deep learning faces many challenges and numerous unsolved problems exist in the research of systemic disease-related ophthalmopathy, its application prospect is bright. With the assistance of AI technology, assistance in clinical diagnosis can be achieved by identifying the unique image markers of each systemic disease-related ophthalmopathy. In addition, the AI image analysis model can also assist in the screening and diagnosis of systemic disease-related ophthalmopathy and may provide clinical screening and medical opportunities for more patients, thus reducing medical costs and medical investment and alleviating the pressure of seeking medical treatment in backward areas.

With the advancement of research, the results of AI application in the field of systemic disease-related ophthalmopathy will greatly improve the level of diagnosis and treatment of systemic disease-related ophthalmopathy to achieve early detection, diagnosis, and treatment of systemic disease-related ophthalmopathy. The application of AI technology in the field of ophthalmology has begun, but on the whole, it is only beginning. As we are entering the era of AI, we should fully utilize AI technology to advance ophthalmology research and make full use of this opportunity and technology to bring about fundamental changes in modern ophthalmology and make AI technology produce more fruitful results in ophthalmology research. This will help in assisting clinicians in the diagnosis and treatment of ophthalmic diseases.

## Figures and Tables

**Table 1:** Summary of DR diagnosis model based on deep learning method.

Study	Task	Sample size	AI model	Output
Gulshan et al. [11]	Identification and detection	EyePACS-1 dataset and Messidor-2 dataset	Deep learning-trained algorithm	On the EyePACS-1 dataset, the AUC value was 0.991, the sensitivity was 90.3%, and the specificity was 98.1%; on the Messidor-2 dataset, the corresponding values were 0.990, 87.0%, and 98.5%.

Ai et al. [12]	Detection	35,126 images	DR-IIXRN	The AUC value and accuracy were 0.95 and 92%.

Bhardwaj et al. [13]	Identification and detection	The DRIVE, STARE, and DIARETDB1 datasets	InceptionResnet-V2	The accuracy was 93.33%.

Li et al. [14]	Detection	35201 images	Deep learning algorithm	The AUC value, sensitivity, and specificity were 0.989, 97.0%, and 91.4%, respectively

Li et al. [15]	Identification	120002 images	The retinal artificial intelligence diagnosis system	The accuracy was 98.1%.

**Table 2:** Summary of HR diagnosis model based on deep learning method.

Study	Task	Sample size	AI model	Output
Abbas et al. [23]	Clinical staging diagnosis	1400 images	DenseNet	The sensitivity was 90.5%, the specificity was 91.5%, the accuracy was 92.6%, the *F*1 score was 92%, and the AUC value was 0.915.

Akbar et al. [24]	Detection and classification	The INSPIRE-AVR and VICAVR datasets and a local dataset	Support vector machine and radial basis function	The average accuracies of the first part were 95.10%, 95.64%, and 98.09%, respectively, and the average accuracies of the second part were 95.93% and 97.50%, respectively.

Arsalan et al. [25]	Detection	The DRIVE, CHASE-DB1, and STARE datasets	A dual-residual-stream-based vessel segmentation network	The sensitivity, specificity, AUC value, and accuracy were as follows: DRIVE: 80.22%, 98.1%, 98.2%, and 96.55%, respectively; CHASE-DB1: 82.06%, 98.41%, 98.0%, and 97.26%, respectively; and STARE: 85.26%, 97.91%, 98.83%, and 96.97%, respectively.

Li et al. [15]	Identification	120002 images	The retinal artificial intelligence diagnosis system	The accuracy was 83.7%.

**Table 3:** Summary of TAO diagnosis model based on deep learning method.

Study	Task	Sample size	AI model	Output
Lin et al. [29]	Detection	160 MRI images of the eyes	A deep learning system based on a deep convolution neural network	The accuracy, sensitivity, specificity, and *F*1 scores of network A were 0.863 ± 0.055, 0.750 ± 0.136, 0.896 ± 0.042, and 0.712 ± 0.121, respectively; the accuracy, sensitivity, specificity, and *F*1 scores of network B were 0.855 ± 0.018, 0.821 ± 0.071, 0.865 ± 0.021, and 0.719 ± 0.040, and the AUC values of the two networks were 0.922.

Song et al. [30]	Detection and identification	1135 orbital CT images	A ResNet-18 derived network	The accuracy, sensitivity, and specificity were 0.87, 0.88, and 0.85, respectively, and the AUC value was 0.919.

Salvi et al. [31]	Classification and progression prediction	242 patients' ophthalmic examination results	A back propagation neural network model with 17 input parameters	The classification accuracy of the model was 78.3%, and the accuracy of predicting progress was 69.2%.

**Table 4:** Summary of NMO diagnosis model based on deep learning method.

Study	Task	Sample size	AI model	Output
Huang et al. [37]	Detection and identification	116 images of magnetic resonance	A multi-parameter multivariate random forest model	In training, the accuracy of the MM-RF model was 0.849, and the AUC value was 0.826; for testing, the accuracy of the MM-RF model was 0.871, and the AUC value was 0.902.

Hagiwara et al. [38]	Detection and identification	53 patients' examination results	SqueezeNet	The AUC value of the model is 0.859, and the accuracies of NMO and MS are 81.1% and 83.3%, respectively.

Kim et al. [39]	Detection and identification	338 patients' images of magnetic resonance	ResNeXt	The AUC value of the model was 0.82, and the accuracy was 71.1%.

Khoury et al. [40]	Identification	202 serum samples	A random forest classification machine learning algorithm	The sensitivity and specificity were 1.00 and 1.00.

**Table 5:** Summary of Behcet's disease diagnosis model based on deep learning method.

Study	Task	Sample size	AI model	Output
The Standardization of Uveitis Nomenclature Working Group [46]	Detection and identification	966 patients	The machine learning method of multinomial logistic regression	The accuracy was 96.3% on the test set and 94.0% on the training set.
Guler and Ubeyli [47]	Detection	The Doppler signals of 106 subjects	A multilayer perceptron neural network model	The accuracy was 96.43% and 93.75%, respectively.

## Data Availability

The datasets used and/or analyzed during the present study are available from the corresponding author on reasonable request.
